# An optoelectronic nose for identification of explosives†
†Electronic supplementary information (ESI) available: Sampling details, handheld reader details, additional array response data, PCA component score plots, ^1^H-NMR of DMDNB and PETN. See DOI: 10.1039/c5sc02632f


**DOI:** 10.1039/c5sc02632f

**Published:** 2015-10-07

**Authors:** Jon R. Askim, Zheng Li, Maria K. LaGasse, Jaqueline M. Rankin, Kenneth S. Suslick

**Affiliations:** a Department of Chemistry , University of Illinois at Urbana-Champaign , 600 S. Mathews Ave. , Urbana , IL 61801 , USA . Email: ksuslick@illinois.edu

## Abstract

Compact and portable methods for identification of explosives are increasingly needed for both civilian and military applications. A portable optoelectronic nose for the gas-phase identification of explosive materials is described that uses a highly cross-reactive colorimetric sensor array and a handheld scanner. The array probes a wide range of chemical reactivities using 40 chemically responsive colorimetric indicators, including pH sensors, metal–dye salts, redox-sensitive chromogenic compounds, solvatochromic dyes, and other chromogenic indicators. Sixteen separate analytes including common explosives, homemade explosives, and characteristic explosive components were differentiated into fourteen separate classes with a classification error rate of <1%. Portable colorimetric array sensing could represent an important, complementary part of the toolbox used in practical applications of explosives detection and identification.

## Abstract 2

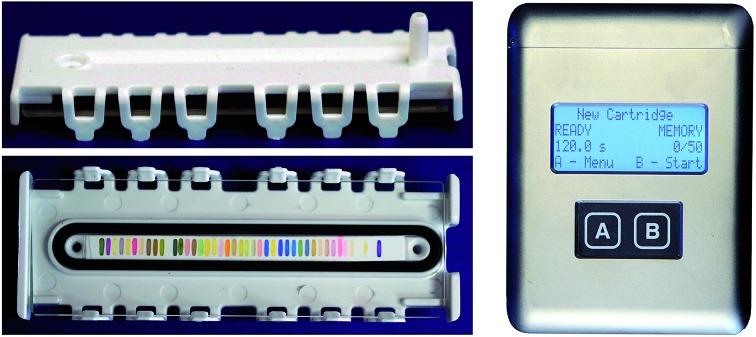
A portable optoelectronic nose for the identification of explosives uses a highly cross-reactive colorimetric sensor array and a handheld scanner.

## Introduction

Intensive research efforts have been made for the detection and identification of explosive compounds. Sensitivity, selectivity, speed, analyte scope, environmental tolerance, device size, and cost all factor heavily into the balance between ideal and practical analysis. Many methods for screening have been investigated, including single-target colorimetric tests,^1^ ion-mobility spectrometry (IMS),^1^,^2^ electronic noses,^3^,^4^ and fluorimetry.^5^,^6^ Despite the breadth of analytical methods available, portable methods still have significant room for improvement: single-target analyses are cumbersome when screening a wide range of analytes; IMS is relatively costly, often requires thermal or ionizing desorption of analytes, and is most useful primarily for nitro-organic detection; and traditional electronic nose technology suffers from sensor drift, poor selectivity and environmental sensitivity (*e.g.*, to changes in humidity or to interferents).^3^,^7^,^8^ In comparison, colorimetric sensor arrays have a broad analyte response, good environmental tolerance, and high selectivity; they are also small, fast, disposable, and can be analyzed using inexpensive equipment.^9^–^11^

Colorimetric sensor arrays combine multiple cross-reactive colorimetric sensors that probe a wide range of analyte chemical properties,^12^–^16^ including Lewis and Brønsted acidity/basicity, molecular polarity, and redox properties. Using a combination of broadly reactive and specifically targeted sensors, colorimetric sensor arrays have been successfully used to differentiate even among similar analytes within diverse families, including toxic industrial chemicals,^12^,^17^ oxidants,^18^ complex mixtures,^19^–^22^ and pathogenic bacteria and fungi.^23^–^25^

**Fig. 1:**
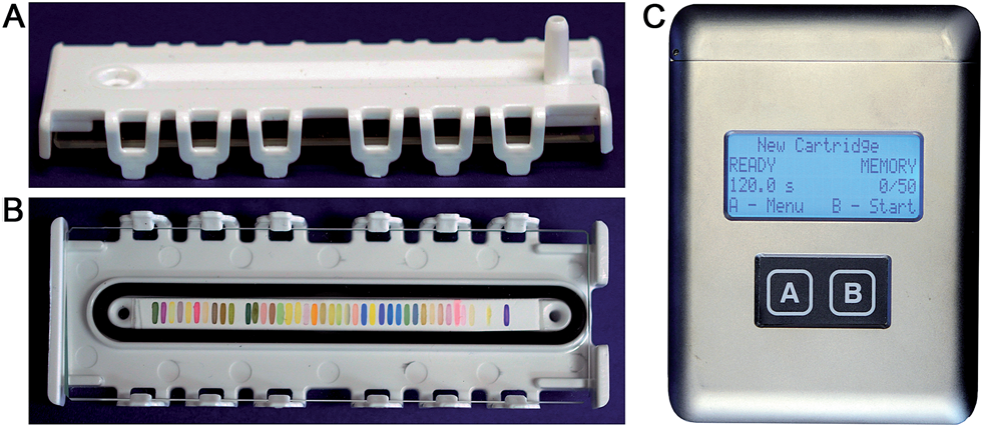
The optoelectronic nose. (A) The linear array of colorimetric sensors and disposable cartridge. Cartridge side view (7.9 × 2.8 × 1.0 cm). (B) Cartridge front view. (C) Handheld reader/analyzer (12.8 × 9.5 × 4.0 cm) based on a color contact line imager.

We report here the development of a new colorimetric sensor array and handheld reader for the identification of explosives and their components. Several new classes of colorimetric sensors were developed including cross-reactive metal–dye salts and other dyes designed to take advantage of the reactivity of carbonyl and nitro compounds. The resulting printed array had forty sensor elements mounted in a snap-together, disposable cartridge (Fig. 1).

**Fig. 2:**
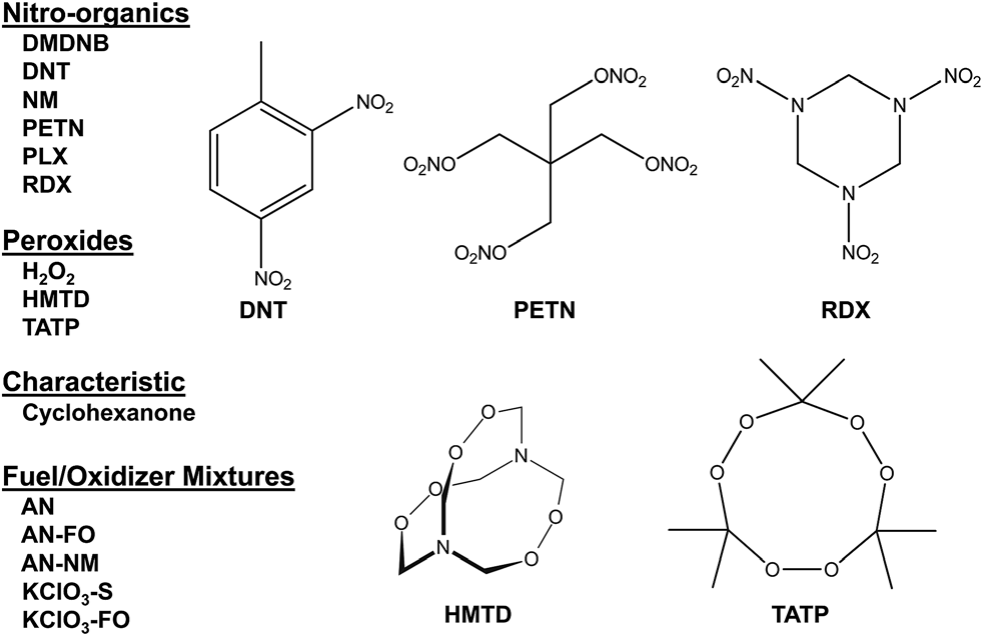
Representative explosives and related compounds targeted for identification using the colorimetric sensor array. Analytes: ammonium nitrate (farm grade, AN), ammonium nitrate/fuel oil (AN–FO), ammonium nitrate/nitromethane (AN–NM), cyclohexanone (C_6_H_10_O), cyclotrimethylene trinitramine (RDX), 2,3-dimethyl-2,3-dinitrobutane (DMDNB), 2,4-dinitrotoluene (DNT), hydrogen peroxide (H_2_O_2_), hexamethylene triperoxide diamine (HMTD), nitromethane (NM), nitromethane/ethylene diamine (Picatinny Liquid Explosive, PLX), pentaerythritol tetranitrate (PETN), potassium chlorate/fuel oil (KClO_3_–FO), potassium chlorate/sugar (KClO_3_–S), and triacetone triperoxide (TATP).

Combined with the colorimetric sensor array, a hand-held reader permits rapid acquisition of low-noise colorimetric data (Fig. 1c). The hand-held reader makes use of a contact image sensor (CIS), a technology commonly used in business card scanners. The careful control of lighting, lack of moving parts, and insensitivity to vibration provides the reader improved signal to noise and faster scan rates compared to other digital imaging techniques;^26^ signal-to-noise ratios in real-time chemical analysis are a factor of 3–10 higher^26^ than currently used methods (*e.g.*, digital cameras, flatbed scanners, smart phones).^13^,^17^,^27^–^30^ We have applied this optoelectronic nose to the identification of sixteen analytes that include common explosives, home-made primary and secondary explosive mixtures, and non-explosive compounds characteristic of military-grade explosives (Fig. 2).^11^,^30^

Compared to dedicated detectors such as those mentioned above, gas-phase sensing using colorimetric sensor arrays has lower sensitivity but significantly broader analyte scope and can identify compounds based on a “chemical bouquet”: *i.e.*, impurities and degradation products that can potentially provide unique signatures. Ultimately, portable colorimetric sensing methods could represent an important, complementary part of the toolbox used in practical applications of explosives detection and identification.

## Results and discussion

### Colorimetric sensor array

Colorimetric sensor arrays make use of multiple cross-reactive dyes in order to give analyte specificity.^9^,^10^ Such arrays are robotically printed and disposable.^9^,^17^ In order to develop a colorimetric sensor array for explosive analytes, several dyes previously found to be broadly cross-reactive (*i.e.*, in discriminating among toxic chemicals,^12^,^17^,^31^ oxidants,^18^ and common organic solvents)^16^ were optimized; these included acid and base-treated pH indicators, Lewis acids, redox-sensitive dyes and chromogens, and solvatochromic dyes formulated with immobilization matrices for printing (Table 1). In addition, several chromogenic species were added to the array to target specific analytes important to the identification of explosives, as discussed below.

**Table 1:** Array composition (top) and color-coded legend of sensor categories (bottom)^*a*^

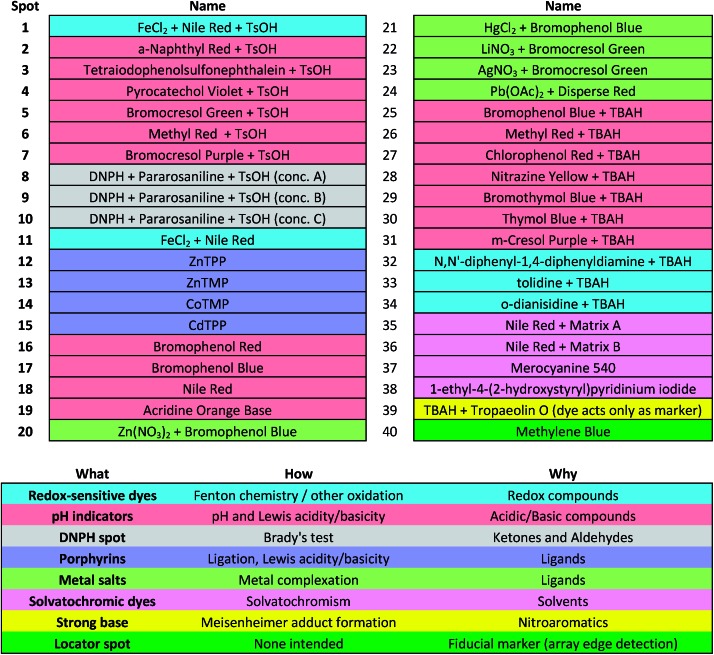

^*a*^TsOH = *p*-toluenesulfonic acid (1 M in 2-methoxyethanol); TBAH = tetrabutylammonium hydroxide (40% in H_2_O); DNPH = 2,4-dinitrophenylhydrazine.

#### Hydrogen peroxide

The array sensor elements responsible for the detection of H_2_O_2_ are based on Fenton's reagent^32^ or on well-established redox indicators.^18^ Acidified ferrous chloride was combined with an empirically-optimized dye (*i.e.*, Nile Red, a very intense, neutral, solvatochromic and pH-responsive dye with a highly conjugated structure) in a printable plasticized medium. When exposed to H_2_O_2_ vapors, the green-blue sensor spot turns yellow-brown as the dye is oxidized by radicals produced catalytically by reaction with Fe^2+^/Fe^3+^.

#### Cyclohexanone

Acidified 2,4-dinitrophenylhydrazine (DNPH), also known as Brady's reagent,^33^ was combined with an empirically-optimized dye (*i.e.*, pararosaniline, a cationic triphenylmethane dye). When exposed to ketones such as cyclohexanone, the red-orange sensor spot turns yellow-brown. There is evidence suggesting that this mixture forms the DNPH analog of Schiff's reagent;^34^ of the several pH indicators tested, only triphenylmethane dyes (*e.g.* methyl violet, crystal violet, pararosaniline, *etc.*) showed any reaction and the color of these dyes changed significantly upon addition of the DNPH reagent, which suggests the specific involvement of reactions between DNPH and the dyes.

#### Nitro-organics

Two strategies were employed for the detection of nitro-organic compounds. First, the use of tetrabutylammonium hydroxide was incorporated into the array for the formation of Meisenheimer complexes with nitroaromatic compounds (2,4-dinitrotoluene);^35^ under the sampling conditions and concentrations used, however, this approach proved to be of limited effectiveness. Second, a new family of chemoresponsive metal/dye complexes was created *in situ* by reaction of the acidic form of dyes and the metal chloride or nitrate salts. Several alkali and transition metal ions were screened; Ag^+^, Li^2+^, Hg^2+^, and Zn^2+^ complexes were found to be the most responsive. Screening of pH indicator dyes revealed bromocresol green (pH range ∼ 3.8–5.4) and bromophenol blue (pH range ∼ 3.0–4.6) to be particularly effective. Further investigation of these salts and the origin of their colorimetric response is ongoing.

### Array response

**Fig. 3:**
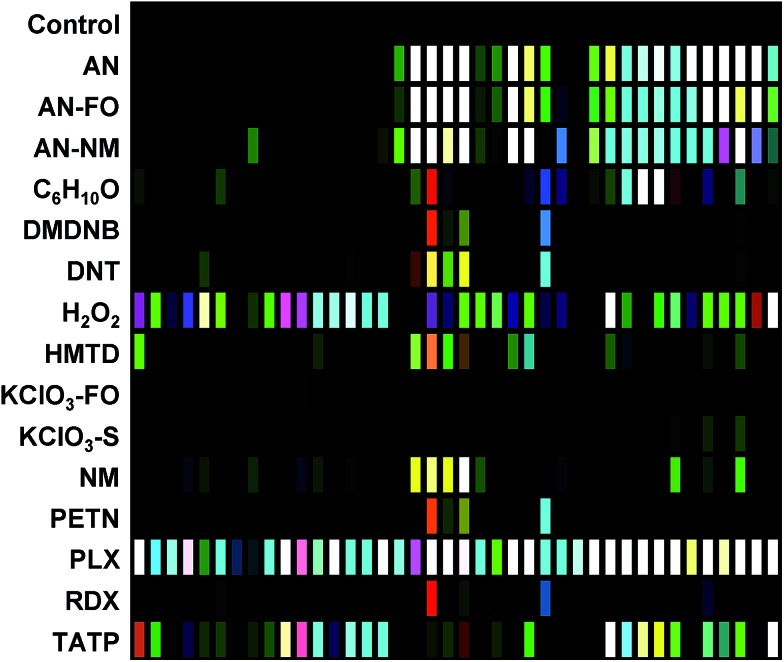
Difference maps of the 40-element colorimetric sensor array showing signal-to-noise of 16 explosives, related analytes, and the control. S/N ratios of 3–10 were scaled for display on an 8 bit RGB color scale (*i.e.*, 0–255).

The digital image of the colorimetric sensor array before exposure is subtracted from that during exposure as described in the Experimental section below. For forty chemoresponsive dyes, this results in a 120-dimensional difference vectors with a total possible value range of –100% to +100% reflectance. Scaled color difference maps of the average signal/noise vectors for each analyte class are shown in Fig. 3 (signal vectors are shown in the ESI, Fig. S4†). These difference maps demonstrate a broad range of response patterns, many of which are discernable even by eye. Among analytes of similar chemical composition, the array responses are similar, but still differentiable in most cases. In general, response magnitude was highly dependent on analyte vapor pressure but also on reactivity: most of the dyes in this chemical sensor array will respond to pH changes, so volatile amines, for example, have a more dramatic response than other volatile but less-reactive species such as cyclohexanone (C_6_H_10_O). It is important to note that many of the analytes have essentially no vapor pressure in pure form (*e.g.*, ammonium nitrate, AN); the array is detecting the volatile components of the analytes that form the “chemical bouquet”, which consists of volatile impurities and degradation products (in the case of AN, for example, these are generally ammonia and amines from the manufacturing process).

Limits of detection (LODs) were determined for the field-appropriate sampling protocol used in these studies and described in the Experimental section below. Air is pulled from a glass vial containing a mg-scale sample into the handheld reader using its internal micropump for 2 min as images of the sensor array were acquired. LODs for this sampling protocol were calculated using the single red, green, or blue response with the highest S/N for AN, NM, and DNT samples (representing highly responsive, moderately responsive, and weakly responsive analytes, respectively) using sample masses ranging from 0.5–100 mg; estimated LODs were as follows: AN = 0.32 mg, NM = 1.31 mg, DNT = 5.19 mg; *cf.* ESI p. S7.† While these mg-scale detection limits are by no means competitive with dedicated trace detectors, they are more than adequate for portable identification of HMEs under field conditions. Improvement in sensitivity by the use of pre-concentrators is of course an option.

### Database evaluation

#### Principal component analysis

Principal component analysis (PCA)^36^–^39^ was employed to provide an estimation of the dimensionality of the data acquired using the colorimetric sensor array, which is itself a measure of the dimensionality of the chemical reactivity space probed by the sensor array. PCA is an unsupervised statistical approach that generates a set of orthogonal vectors (*i.e.*, principal components) using a linear combination of array response vectors to maximize the amount of variance into the fewest number of principal components. The resulting principal components are *not* optimized for analyte discrimination: PCA describes the entire dataset using components to capture the maximum amount of total variance; this does not necessarily maximize discrimination ability among classes of analytes.

**Fig. 4:**
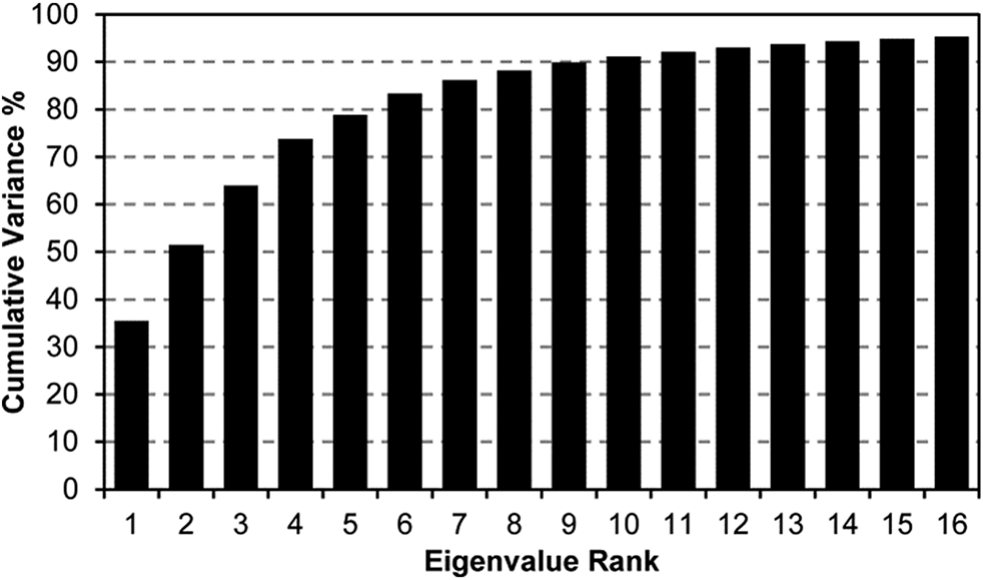
Scree plot of the principal component analysis for 15 explosives and related compounds. 16 dimensions were required to capture 95% of the total variance, consistent with the colorimetric sensor array probing a wide range of chemical reactivity.

A scree plot of the normalized data collected for explosives analytes is given in Fig. 4. A total of 10 dimensions were required to capture 90% of the total variance and 16 dimensions for 95%; such high dimensionality is consistent with the very broad range of chemical reactivities being probed by the colorimetric sensor array, as we have noted before with other analytes.^9^,^19^,^23^,^31^ The high dimensionality of the colorimetric sensor array is in stark contrast to traditional electronic nose technology in which only 1 or 2 dimensions are required to reach 95% of the total variance (in these cases, the sensor array is probing only a very limited range of chemical properties, *e.g.*, hydrophobicity/surface adsorption).^9^

When using PCA to discriminate among analytes, one typically plots the data points using the first two (or, rarely, three) principal components in a score plot. The assumption in these cases is that the vast majority of the discrimination ability is contained in these first few principal components. This is only true, generally, when the first few principal components also describe the vast majority of the total variance. The high dimensionality of the colorimetric sensor array data, however, makes PCA generally ill-suited for use in discrimination among analyte species: too little of the variance is captured in two or three dimensions to avoid overlap of analyte classes, and in fact, PCA score plots show significant overlap among classes even among samples that show obvious qualitative differences in response (ESI, Fig. S6†).

#### Hierarchical cluster analysis

**Fig. 5:**
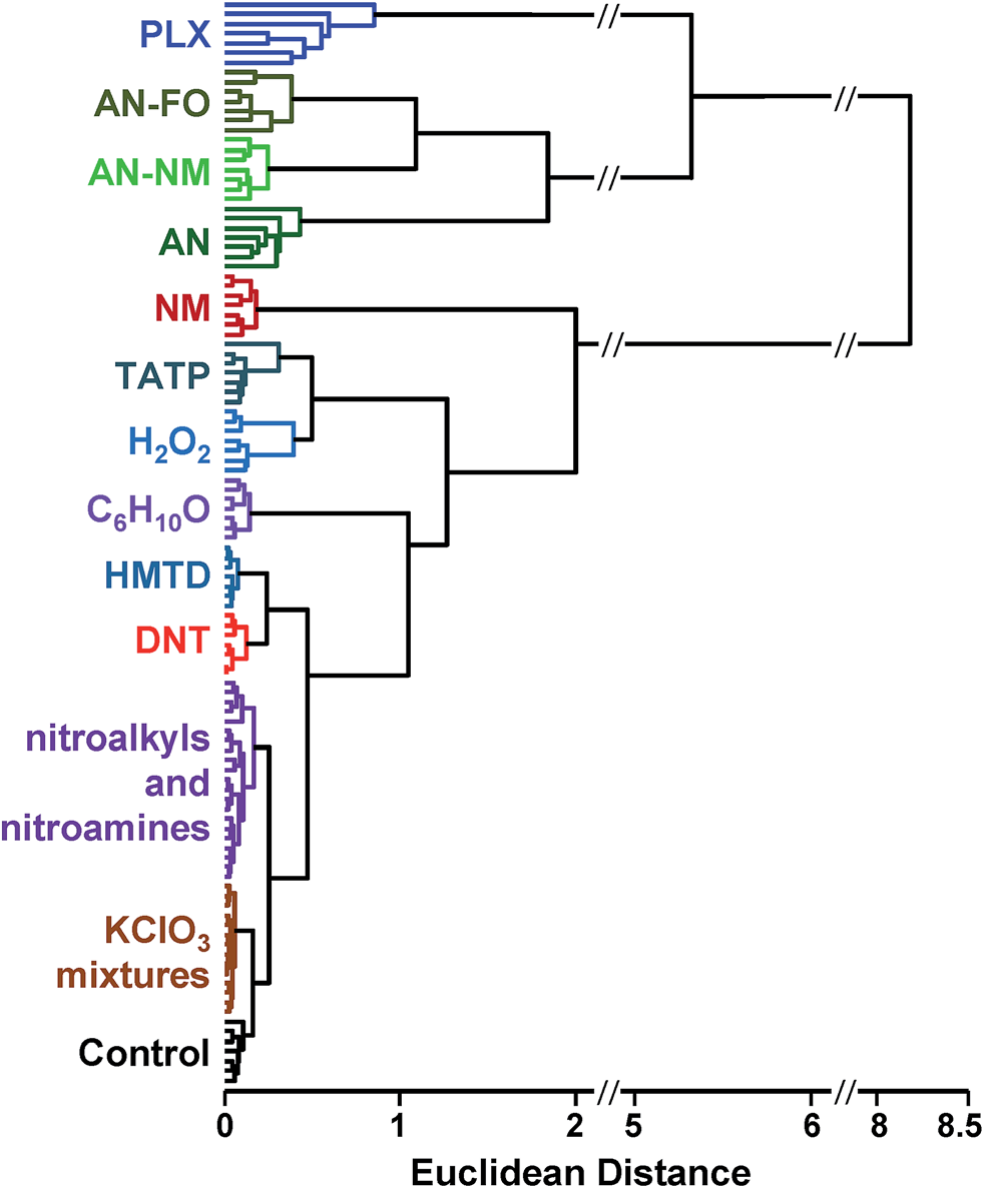
Hierarchical cluster analysis (HCA) dendrogram of the normalized difference vectors (*i.e.*, changes in reflectance) for 16 explosives, related analytes, and the control; 112 trials in total. All species were clearly differentiable except among members of two groups: KClO_3_ mixtures (KClO_3_–sugar and KClO_3_–fuel oil) and nitroalkyls/nitroamines (DMDNB, PETN, and RDX).

Hierarchical cluster analysis (HCA)^36^,^38^ was used to give a model-free evaluation of the acquired database and the relative similarities among the data collected. HCA is an unsupervised clustering technique that groups array response data in the full 120-dimensional vector space (*i.e.*, color difference changes in red, green, and blue for each of the 40 sensor elements on the colorimetric sensor array); starting with single data points, clusters are formed hierarchically by connecting the centroids of unconnected clusters or data points (in this case using Ward's method, which minimizes the total within-cluster variance). The resulting dendrogram shows connectivity (indicating which clusters are most similar to each other), and inter-cluster distance (describing the magnitude of dissimilarity between clusters). The HCA dendrogram for the response of these common explosives is shown as Fig. 5. The method shows obvious discrimination among 11 of the 16 analytes (including the control). Confusions of clustering were observed among two groups: that containing the weakly-responding potassium chlorate mixtures (KClO_3_–fuel oil and KClO_3_–sugar) and separately, the group containing nitroalkyls and nitroamines (PETN, RDX, and DMDNB).

#### Customized HCA or PCA algorithms

If one were insistent upon using HCA or PCA for discrimination, it is possible to use additional information (such as principal components or cluster centroids) to construct a high-dimensional space, and to develop a supervised algorithm to determine the minimum amount of information (*e.g.*, the number of principal components) required to discriminate with some arbitrary level of accuracy. Doing this, however, defeats the purpose of using HCA or PCA: these methods are fast, simple analytical tools that work well for initial determination of the quality of a dataset. There are better choices for determining discrimination ability in high-dimensional space, such as support vector machines, as described below.

### Classification methods

Classification methods involve developing *classifiers* – algorithms that can predict the identity of an unknown compared to an established database (*i.e.*, library or training set). Classifiers are based on a decision boundary by which an incoming sample can be classified, for example “Analyte A *vs.* Analyte B” or “Analyte C *vs.* not Analyte C”. Of necessity, such classifications are supervised methods: the identity of the samples in the database must be known. While unsupervised methods cannot be used to classify data, they can be used as the first step to develop the decision boundaries.

HCA and PCA are unsupervised methods used to analyze an existing data set. Because they are unsupervised, they provide no direct method for class prediction from data on an unknown sample (*i.e.*, classification). Although HCA and PCA do not present a direct method for classification, they can be used to develop decision boundaries if clustering is completely free of errors/confusions. As described above, however, these decision boundaries are not optimized to give the best discrimination ability: HCA clusters based on a cascading nearest-neighbor method, while PCA develops principal components based on maximizing the variance among all points in the dataset. Rather than attempt to use these non-optimized methods to develop classifiers, we chose to use a common machine learning tool that was specifically developed to maximize discrimination ability: support vector machines (SVM).^40^

#### Support vector machines

**Fig. 6:**
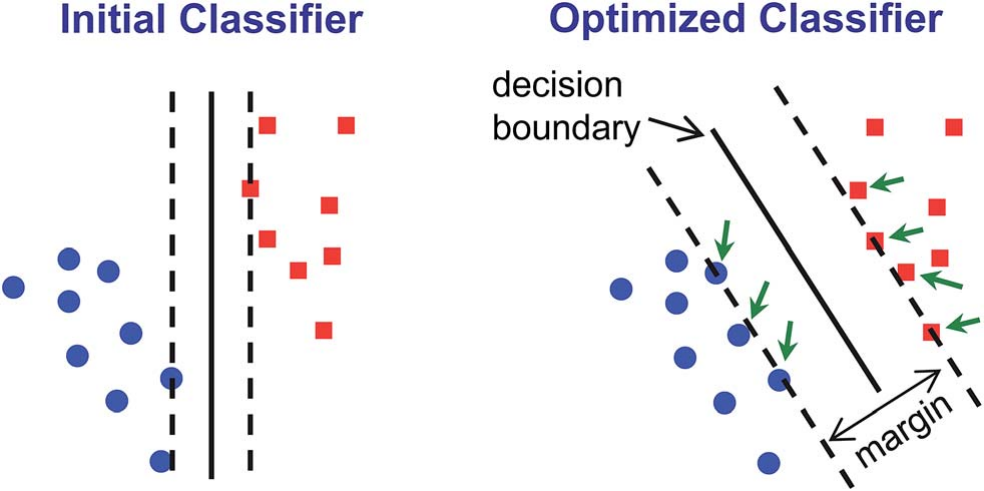
Graphical illustration of SVM classifier optimization. A simplified initial guess is performed (left) and then algorithmically optimized through multiple iterations to maximize discrimination ability (typically, by maximizing the size of the margin and minimizing offside errors, as shown on the right). The margin is defined as the distance from contentious points (*i.e.*, support vectors, indicated by green arrows) to the decision boundary.

Unlike unsupervised methods such as PCA, HCA, or other clustering methods, SVM is a predictive method that is designed to classify incoming data that is not part of the training database. SVM classification is based on pairwise class prediction and focuses on the data most likely to be misclassified (*i.e.*, data vectors near the decision boundary for any given class pair, the so-called support vectors) to create optimized decision boundaries that best separate the data for each given pair of classes in high dimensional space. The result of each pairwise comparison gives a vote that is used to determine the final classification.^40^ A general graphical explanation of the process is shown in Fig. 6. SVM optimization factors have been fully developed and incorporated into LIBSVM, an open-source SVM library.^41^

SVM is well optimized for discrimination within multidimensional datasets and has been widely and successfully developed, for example, for identification of objects in machine vision. Implementation of SVM uses multiple rounds of iteration to optimize parameters. Typical use involves data transformation using a kernel to convert the dataset into a linearly separable arrangement (*i.e.*, data lie on one side of a plane or the other, but not both). Using the colorimetric sensor arrays, the class data were found to be roughly normally distributed and linearly separable; no data transformation was necessary (*i.e.*, a linear kernel was used). Default SVM parameters were found to be quite well-optimized for the colorimetric sensor array database; this is not surprising, since HCA results already showed a high level of separation using a Euclidean distance clustering method.

Classifiers for each pair of analyte groups took the form of a decision hyperplane defined by an orthogonal 120-dimensional vector (*i.e.*, optimized linear combinations of ΔRGB values of the sensor array) combined with a scalar value marking the position of the decision boundary; implementation in the automated handheld platform was simple, as it only required projection of the incoming 120-dimensional data vector from each scan onto the classifier vector using an inner product and comparison to the decision boundary scalar.

#### Classification accuracy

Classification accuracy of the SVM method was estimated using a leave-one-out cross-validation method. The database was divided into training sets and evaluation sets in a permutative manner: classifiers are created based on training sets (*i.e.*, with one trial left out) and predictions then made on the evaluation sets (*i.e.*, the left-out trial) and iterated among all permutations. Cross-validation results are shown in Table 2. For 12 of the analytes, no errors during cross-validation were observed among the septuplicate trials; the two KClO_3_ mixtures (KClO_3_–sugar and KClO_3_–fuel oil) and two nitro-organics (DMDNB and PETN), however, were non-separable within their respective groupings. In comparison to HCA, SVM was able to completely differentiate RDX (a nitroamine) from DMDNB and PETN (a nitroalkane and an alkyl nitrate ester, respectively).

**Table 2:** Support vector machine (SVM) classification results (*i.e.*, leave-one-out cross-validations of 112 permutations) of 16 common explosives, related compounds, and the control. The accuracy shown for each analyte represents the percentage of correctly identified analytes among 7 independent trials. Similarly, the misidentifications indicate which incorrect classifications were supplied by the algorithm. Consequently, the KClO_3_ analytes should be considered a single group of analytes, and likewise, DMDNB and PETN

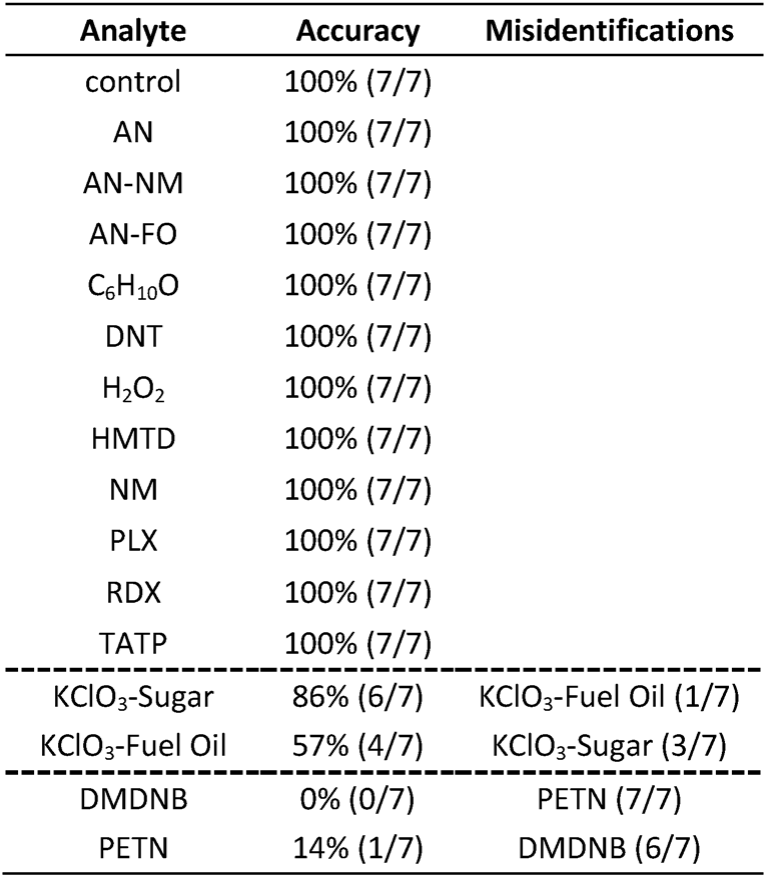

In the case of the KClO_3_ mixtures, both analytes give very low array response, but all individual trials were still distinguishable from the control group. None of the components (*i.e.*, KClO_3_, sucrose, or corn starch, which is the primary additive in commercial sugar) are sufficiently volatile to provide a strong response and the sensor array is known to be relatively insensitive to hydrocarbons.^42^ Confusions among the chlorate analytes are therefore unsurprising. Although in these trials we find no confusions between the chlorate analytes and the control samples, we are not fully confident in the colorimetric sensor array's ability to detect these chlorate species: the maximum signal in all KClO_3_ mixture samples barely exceeds estimated detection requirements (maximum observed S/N ≈ 9 for chlorate analytes, whereas the estimated detection limit requires S/N ≈ 7–9, *cf.* ESI p. S7†). In addition, the species to which the array is responding for the apparent detection of KClO_3_ mixtures and discrimination from control samples remains an open question.

In the case of the nitro-organic species, DMDNB and PETN were not separable in our sensor array analysis. ^1^H-NMR showed that the DMDNB and PETN samples did not share any detectable components down to concentrations as low as 0.02 mol%, which makes it unlikely that the similarity of array response for both analytes is due to the same trace compound or contaminant (ESI, Fig. S7†). The similarity in array response is then likely due to the similar chemical reactivity of the two analytes, *i.e.*, the array does not distinguish between these two nitro-organics because their chemical reactivities are so similar.

If one re-analyzes all the data with 14 classes (*i.e.*, grouping chlorate mixtures and separately grouping DMDNB with PETN), SVM accuracy is raised to 100%. The groupings of chlorate analytes and of two nitro-organics does not diminish the utility of the method: KClO_3_ is the relevant component in its explosive mixtures (it will react with essentially any oxidizable material). Similarly, both DMDNB and PETN are found in similar explosives materials; DMDNB is commonly used as a volatile taggant (at 0.05 wt%) for explosives containing the much less volatile PETN and RDX.^8^,^43^,^44^

## Conclusions

A colorimetric sensor array was developed for classification of common explosives and related compounds relevant to home made explosives (HMEs) and improvised explosive devices (IEDs). The array incorporates acid–base indicators (both Lewis and Brønsted), metal–dye salts, redox-sensitive chromogenic compounds, solvatochromic dyes, and other reactive chromogenic mixtures designed to take advantage of the unique reactivity of carbonyl and nitro compounds; this results in a highly cross-reactive sensor array capable of probing a very wide range of chemical reactivities. RGB (red, green, blue) color values were collected with a custom-designed handheld reader/analyzer making use of a linear color contact image sensor; the handheld unit is capable of automated classification analysis without operator input. Based on cross-validation results, support vector machine analysis was able to discriminate 16 separate analytes into 14 groups with an estimated accuracy approaching 100%. This method has significant implications in portable explosives identification and may prove to be a useful supplement to other current technologies.

## Experimental section

### Colorimetric sensor arrays

The colorimetric sensor arrays were printed on polypropylene membranes (0.2 μm) purchased from Sterlitech Corporation (Kent, WA, USA). All reagents were analytical-reagent grade, purchased from Sigma-Aldrich and used without further purification. Cartridges were custom injection-molded using low-volatility white polycarbonate (Dynamic Plastics, Chesterfield Twp, MI, USA). The polypropylene membrane strips were adhered to cartridges using low-volatility 3M™ 465 Adhesive Transfer Tape (3M Co., St. Paul, Minnesota, USA). Colorimetric sensor arrays were robotically printed on these substrates using procedures described previously;^17^ in this case, custom-designed rectangular pins were used instead of round pins, and 40 spots were printed at 1.2 mm center–center distance; images of the pins used are shown in ESI, Fig. S1.† The chemoresponsive dyes used in each spot are described in Table 1 along with a color-coded legend indicating the intended purpose of each spot (*i.e.*, expected chemical reactivity).

### Handheld reader

The portable reader used in this work uses a compact color line imager (contact image sensor, or CIS) combined with a linear array of chemoresponsive dyes. CIS operation is shown schematically in ref. 45. Internal images of the handheld reader and its specifications are provided in the ESI, Fig. S2 and Table S1.† As indicated, the analyte gas flow path is directly from the sample container into the cartridge over the sensor array and out through a diaphragm micropump (Schwarzer SP100-ECLC), which minimizes the possibility of cross-contamination. Illumination levels for the RGB LEDs were controlled using a combination of applied voltage and pulse-width modulation. Raw data were normalized using a calibration created from a one-time measurement of a 0% reflectance standard (*i.e.*, the sensor array with all LEDs turned off) and a 100% reflectance standard (*i.e.*, a blank array without any printed sensor elements).

### Analyte samples

All reagents were purchased from Sigma and used without purification except as follows: generic farm-grade/commercial-grade ammonium nitrate (AN) was purchased from Fredrich Electronics (Boonville, MO). RDX and PETN were supplied by Los Alamos National Labs (Los Alamos, NM). TATP and HMTD were synthesized as described elsewhere^46^,^47^ on reduced scale (<100 mg). **Caution: TATP and HMTD are extremely sensitive explosives!** Fuel oil was purchased as diesel fuel from a local gas station. Powdered sugar was purchased from a local market. Detailed compositions of these analytes are described in the ESI, Table S2.†

### Sampling protocol

Samples were prepared by weighing 100 mg of each analyte into 7 mL snap-top polypropylene scintillation vials; fuel-oxidizer mixtures were prepared based on a 1 : 1 stoichiometric ratio. In order to reduce risks during synthesis and storage, 20 mg samples were used for TATP and HMTD. **Caution: Do not use screw cap vials; powder left on the screw threads are an explosion hazard when caps are screwed down.**

Analytes were tested using a field-appropriate sampling protocol; milligram-scale samples (100 mg for most analytes, 20 mg for HMTD and TATP) were stored in small glass vials and the headspace was sampled through a short Teflon tube while open to the ambient environment. Arrays were imaged with a handheld reader/analyzer (ESI, Fig. S2 and S3†) that contains an optical line imager (12 bit contact image sensor, CIS). Using the onboard micropump, arrays were initially exposed to control media (ambient lab air, ≈30% relative humidity at 24 °C) for 2 minutes and a ‘before exposure’ image acquired by the handheld imager. Arrays were then exposed to analyte head space by pumping air from sample vials using a short Teflon feed tube (3.8 cm) through the sensor cartridge for 2 minutes and an ‘after exposure’ image acquired. Analyte response was calculated from the difference between the measured red, green, and blue (RGB) values before and after exposure (*e.g.*, Δ*R* = *R*_after_ – *R*_before_). Seven independent trials were run for each analyte sample using separate arrays.

Difference maps (which are used only for visualization) were constructed by taking the absolute value and scaling a relevant color range (indicated on each difference map) to the 8-bit color scale (*i.e.*, 0–255). For S/N measurements, signal and noise were calculated for each ΔRGB dimension using all trials in the control data set (*i.e.*, red, green, and blue values for each sensor element; total of 120 dimensions for an array with 40 sensor elements); the signal for each dimension was defined as the difference between each analyte trial measurement and the control average (*e.g.*, Δ*R*_analyte-*n*_ – Δ*R*_control-avg_) and noise was defined as the standard deviation in the control data set (*e.g.*, *σ*_R_^2^ = ∑_*n*_(Δ*R*_control-*n*_ – Δ*R*_control-avg_)^2^/(*N* – 1)).

### Database analysis and classification

Hierarchical cluster analysis (HCA) was performed using Ward's method (*i.e.*, total Euclidean distance variance minimization) with Matlab software (MathWorks Inc., Natick, MA, USA). Principal component analysis (PCA) was performed using MVSP software (Kovach Computing Services, Pentraeth, Isle of Anglesey, UK). Support vector machine (SVM) analysis was performed using custom software making use of LIBSVM, an open source SVM library, using a linear kernel with default parameters.^41^

## Supplementary Material

Supplementary information
