# Supplementary material for: An optoelectronic nose for identification of explosives
Source: Chem Sci. 2015 Oct 7;7(1):199–206. doi: 10.1039/c5sc02632f (PMC5952316; doi:10.1039/c5sc02632f)
Supplement: Supplementary file 1 [file SC-007-C5SC02632F-s001.pdf]

Electronic Supplementary Material (ESI) for Chemical Science.
This journal is © The Royal Society of Chemistry 2015

Electronic Supplementary Information

An optoelectronic nose for identification of explosives

Jon R. Askim, Zheng Li, Jacqueline M. Rankin, Maria K. LaGasse, and Kenneth S. Suslick*

Department of Chemistry, University of Illinois at Urbana-Champaign, 600 S. Mathews Ave., Urbana, IL 61801, USA.

*To whom correspondence should be addressed: Prof. Kenneth S. Suslick, Department of Chemistry, University of Illinois at Urbana-Champaign. 600 S. Mathews Ave. Urbana, IL 61801, USA. Tel: 1-217-333-2794. Email: ksuslick@illinois.edu

Table of Contents

Item	ESI Page
Array Preparation	S3
Figure S1. Pin holder for array printing	S3
Handheld Reader Details	S3 - 4
Figure S2. Handheld reader photographs	S3
Table S1. Handheld reader component parameters	S4
Analyte Sample Details	S4
Table S2. Analyte composition	S4
Sampling Procedure	S5
Figure S3. Sampling setup photographs	S5
Figure S4. Raw difference image example	S5
Figure S5. Difference maps for all analytes	S6
Limits of Detection	S7
Principal Component Analysis	S8
Figure S6. PCA score plot	S8
1H-NMR spectra of DMDNB and PETN	S9
Figure S7. 1H-NMR spectra of DMDNB and PETN	S9
ESI References	S10

Array Preparation

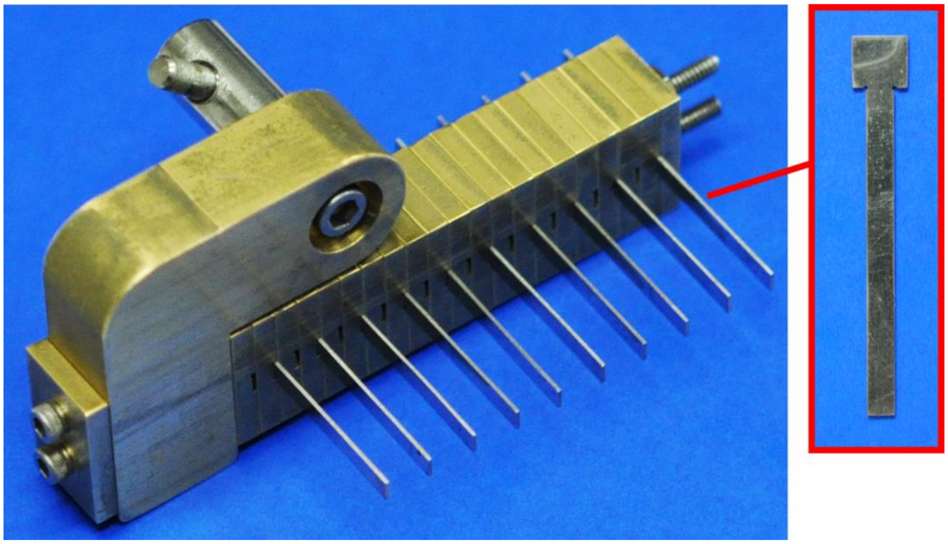

Figure S1. Pin holder for rectangular pins used to print linear colorimetric sensor arrays (left) and close-up view of a rectangular pin (right).

Handheld Reader Details

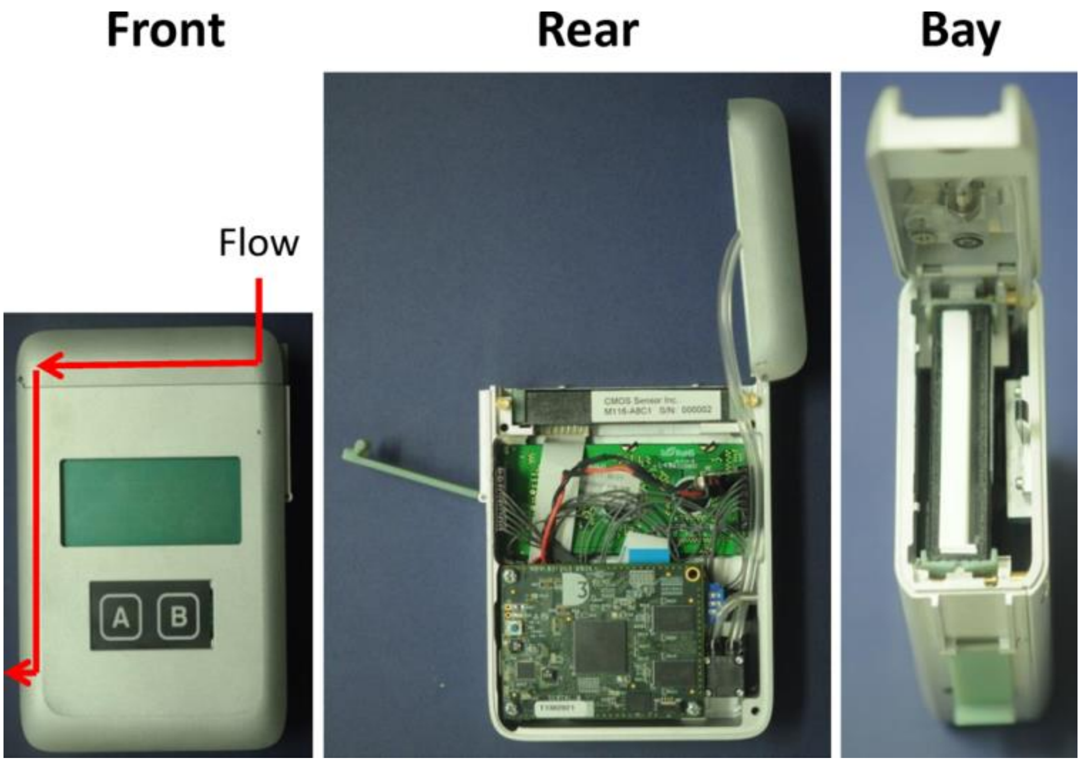

Figure S2. Photographs of the handheld reader including front, rear, and cartridge bay views. Dimensions are 12.5 cm tall by 9.5 cm wide by 4.0 cm thick. The rear panel and 9 V battery were removed in order to provide a better view of the internal electronics and diaphragm micropump (located in rear image, lower right).

Table S1. Relevant component parameters of the handheld reader.

Scanner Size	12.8 cm x 9.5 cm x 4.0 cm
Scanner Weight	460 g + battery + cartridge
Cartridge Size	7.9 cm x 2.8 cm x 1.0 cm
Cartridge Weight	11 g
Battery Weight	48 g
Static Pressure	550 mbar typical
Pump Rate	580 cm3/min typical
Current Draw	~400 mA
Battery Charg	1200 mAh typical
Samples/Battery	100 typical

Analyte Sample Details

Table S2. Composition of all explosive analytes and related compounds.

Name	Component(s)
control	Ambient lab air (approx. 30% rel. humidity at 24°C)
AN	Ammonium Nitrate, farm grade (FG)
AN-FO	94.48 wt% Ammonium Nitrate, FG + 5.52 wt% Fuel Oil (diesel)
AN-NM	66.30 wt% Ammonium Nitrate, FG + 33.70 wt% Nitromethane
$C_6H_{10}O$	Cyclohexanone
DMDNB	2,3-Dimethyl-2,3-dinitrobutane
DNT	2,4-Dinitrotoluene
H_2O_2	Hydrogen Peroxide (30% in H_2O)
HMTD	Hexamethylene Triperoxide Diamine
NM	Nitromethane
PC-S, $KClO_3$-S	74.12 wt% Potassium Chlorate + 25.88 wt% Powdered Sugar
PC-FO, $KClO_3$-FO	89.73 wt% Potassium Chlorate + 10.27 wt% Fuel Oil
PETN	Pentaerythritol Tetranitrate
PLX	95.00 wt% Nitromethane + 5 wt% Ethylene Diamine (aged 24 hours)
RDX	Cyclotrimethylenetrinitramine
TATP	Triacetone Triperoxide

Sampling Procedure

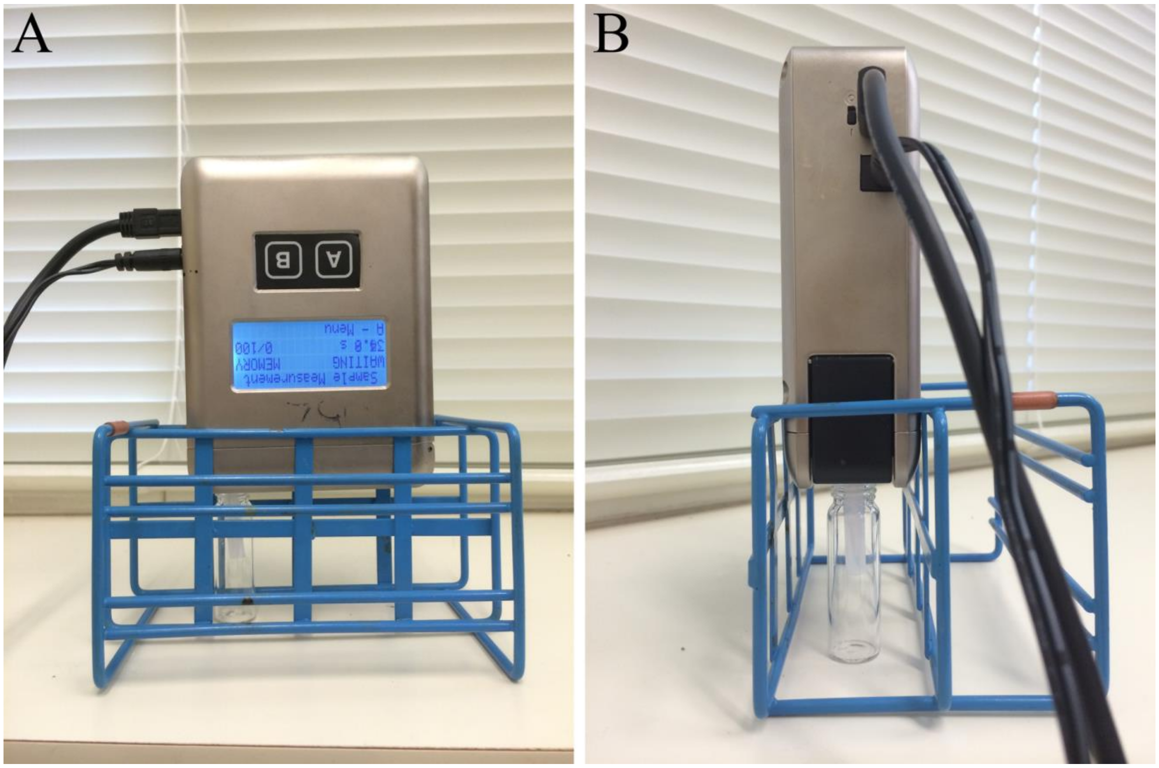

Figure S3. Photograph of sampling setup. The handheld device was held in a metal rack with a tube inserted into a glass vial containing the analyte, shown from the (A) front, and (B) side. The array cartridge is attached to a short feed tube (3.8 cm). Prior to analyte headspace sampling, the array is equilibrated to the ambient background atmosphere for 2 minutes. The feed tube is then inserted into a 7 mL glass vial for headspace sampling, and measurements were collected after 2 minutes of exposure to sample headspace at a flow rate of approximately 580 cm^3/minute (sccm).

One example showing a graphical form of subtraction on raw images is shown as Figure S4; note that this was figure was generated by subtracting the numeric values and constructing a bitmap for visualization. Average difference maps for each analyte class and the control are shown in Figure S5. As shown, the array responses vary considerably across the range of tested analytes.

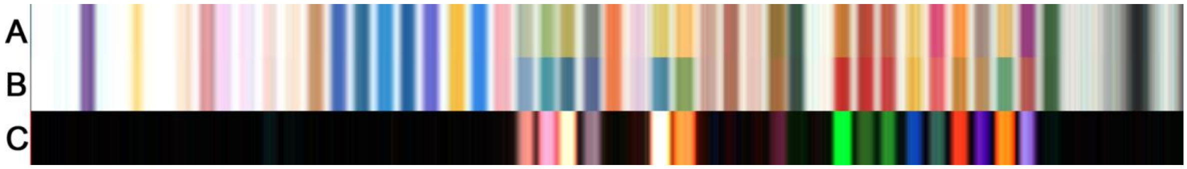

Figure S4. Raw sensor images collected before (A) and after (B) exposure to AN head gas, and a scaled difference map (C, RGB color range 0-16 scaled to 0-255).

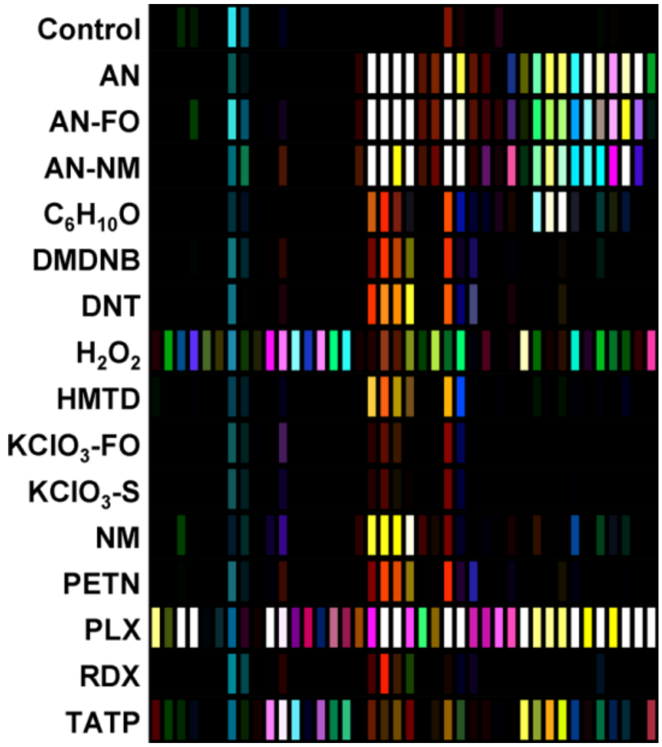

Figure S5. Average sensor response vectors of 15 explosives and related compounds. Normalized reflectance values were scaled so that the relevant response range (i.e., 0.39% to 3.9% of maximum reflectance) is displayed on an 8-bit RGB color scale (i.e., 0-255).

Limits of Detection

The limits of detection (LODs) for this sampling protocol were estimated for AN, NM, and DNT samples (representing highly responsive, moderately responsive, and weakly responsive analytes respectively) using sample masses ranging from 0.5-100 mg; calculated LODs were as follows: AN (0.13 mg), NM (0.52 mg), DNT (1.8 mg).

The limit of detection for this dataset was defined as the 99% confidence threshold using the single dimension with the highest signal-to-noise ratio. Each dimension S_N is individually approximately Gaussian-distributed and not necessarily independent (i.e., covariance is significant). The function defining the detection limit is $S_{max} = Max(S_N)$, which is not Gaussian distributed; theoretically, the distribution is a form of the Error function that is well beyond the scope of this work. Further complicating the problem is the fact that the true standard deviation for the control data is not known; we instead estimate the standard deviation using a t-distribution with 6 degrees of freedom (i.e., 7 experimental trials for the control). To approach this problem, we created a simulation in which we calculated S_{max} many times (10,000 trials) for 120 independent trials using the appropriate t-distribution and determined a 99% confidence threshold. For a completely dependent dataset (i.e., in which all 120 dimensions are scalar multiples of a single dimension), the probability distribution of S_{max} reduces down to the probability distribution of S_N, which has already been estimated as a t-distribution with 6 degrees of freedom. Therefore, the detection limit threshold is somewhere between the fully-dependent value and the fully-independent value: $S_{max} = 3.707$ to $S_{max} = 9.080$. Based on the scree plot from the PCA data, approximately 95% of the total variance was captured in 16 dimensions; therefore, we chose to instead use 16 independent t-distributed dimensions to develop a 99% confidence threshold. This lead to a value where $S_{max} = 6.28$; coincidentally, this is very close to halfway between the thresholds estimated for the fully-dependent and fully-independent values.

Limits of detection were then calculated as follows. Response values for a control sample were collected (seven independent trials). Response values were also collected in triplicate trials at several different sample masses: AN: 0.5 mg, 1 mg, 2 mg, 5 mg, 10 mg, 20 mg; NM: 2 mg, 5 mg, 10 mg, 20 mg, 50 mg; DNT: 5 mg, 10 mg, 20 mg, 50 mg. A single-point LOD was then calculated for each trial and dimension using LODsingle = Mass * 6.28 * StDevControl / (ResponseTrial - ResponseAvgControl); the calculated LOD for a trial is then the maximum LODsingle among all dimensions for the trial. Plotting LOD vs. sample mass, second-order least-squares interpolation gave polynomials of the form $Ax^2 + Bx + C$ with the following R^2 values (AN = 0.8675, NM = 0.9864, DNT = 0.9755). These 2nd order polynomials were then solved for y = x, corresponding to the position where the calculated LOD was equal to sample mass (AN = 0.32 mg, NM = 1.31 mg, DNT = 5.19 mg). Note that single-point LOD estimates calculated using the lowest tested sample mass (AN: 0.5 mg, NM: 2 mg, DNT: 5 mg) deviated only slightly from the values calculated using quadratic fitting (AN = 0.39 => 21% higher, NM = 1.53 => 17% higher, DNT = 4.83 => 7% lower).

Principal Component Analysis

As shown in the main text (Figure 4), a large number of principal components were required in order to adequately describe the dataset (i.e., 16 dimensions are required to capture greater than 95% total variance). In order to demonstrate how this translates into discrimination ability (or lack thereof) by PCA two-dimensional score plots and to compare this sensor array to other works that rely on low dimensional data and PCA for analysis, the first four principal components were plotted as shown in Figure S6. Upon cursory examination, it is obvious that PCA using the first two dimensions provides adequate discrimination for some analytes (e.g., AN, AN-NM, AN-FO, NM, etc) but shows essentially no discrimination for others (e.g., both nitroalkanes, both chlorate-containing analytes, and HMTD all appear to be inseperable using the first two dimensions). Extending this to the third and fourth dimensions, additional analytes are able to be discriminated (TATP, H2O2, cyclohexanone, etc) but there is still significant overlap among many analytes. High dimensionality data is not easily interpreted by two-dimensional graphs, which is why more sophisticated analyses (e.g., HCA and SVM) are required for colorimetric sensor array data.

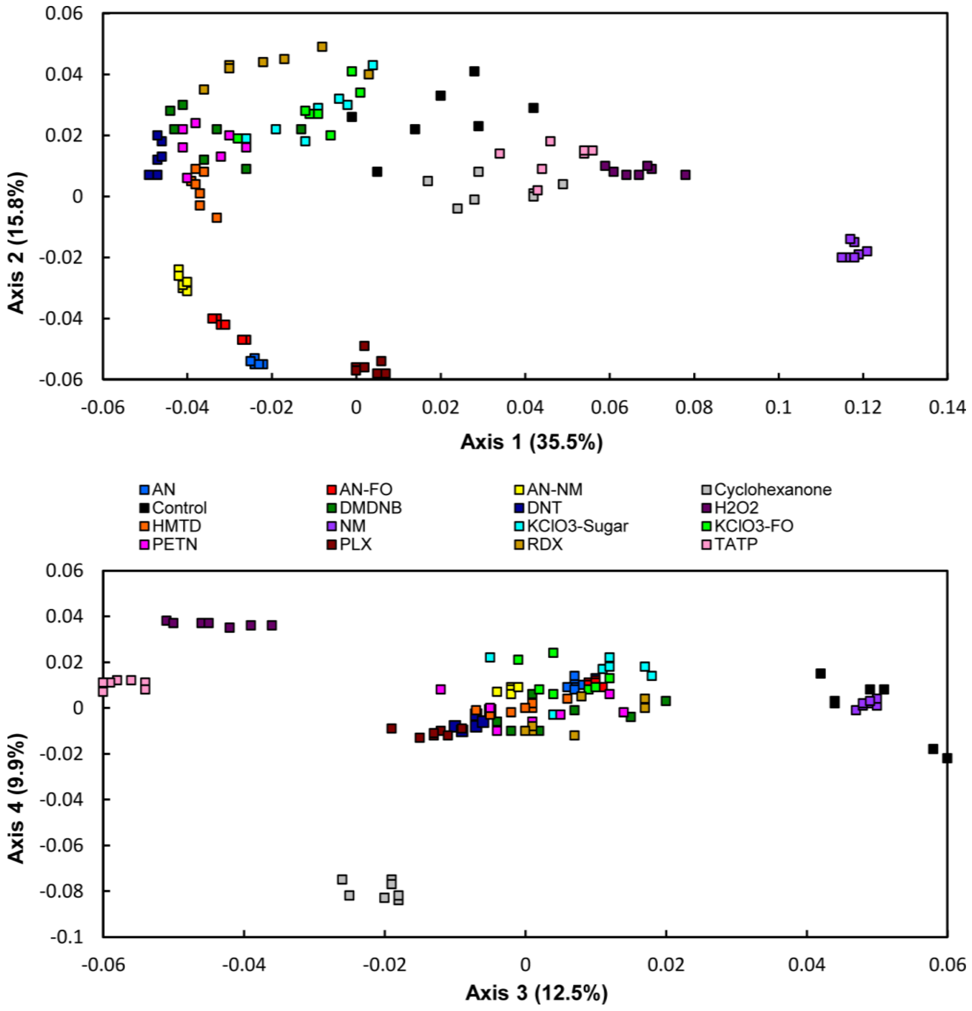

Figure S6. PCA two-dimensional score plots for the first and second and for the third and fourth principal component axes. Two dimensional plots of the first four components show essentially no discrimination among several analyte classes (e.g., nitroalkanes, chlorate-containing species, HMTD). This reflects the very high dimensionality of the colorimetric sensor array data: the first four dimensions contain only 35.5%, 15.8%, 12.5%, and 9.9% of the total variance, respectively.

^{1}H-NMR spectra of DMDNB and PETN

^{1}H-NMR spectra of DMDNB (2,3-dimethyl-2,3-dinitrobutane) and PETN (pentaerythritol tetranitrate) were collected with a Varian 500 MHz NMR spectrometer with a narrow-bore coil (Varian Inc, Palo Alto, CA, USA). The limit of detection of this method was defined as the point at which peaks could no longer be resolved from baseline noise (i.e., 3*100 mol % / $\sigma_{baseline}$).

Since DMDNB and PETN were unable to be distinguished from each other, it was suspected that the PETN sample might have contained significant amounts of DMDNB (which is commonly used as a taggant for PETN and other explosives). However, NMR analysis conclusively disproved this hypothesis. The absence of any detectable DMDNB peak in the PETN spectrum (Figure S7) indicates that the amount of DMDNB present in the PETN sample is less than the detection limit of the method, which was estimated to be approximately 0.02 mol %. This detection limit is slightly below the concentration expected for a standard taggant (~ 0.04 mol %).[1-3]

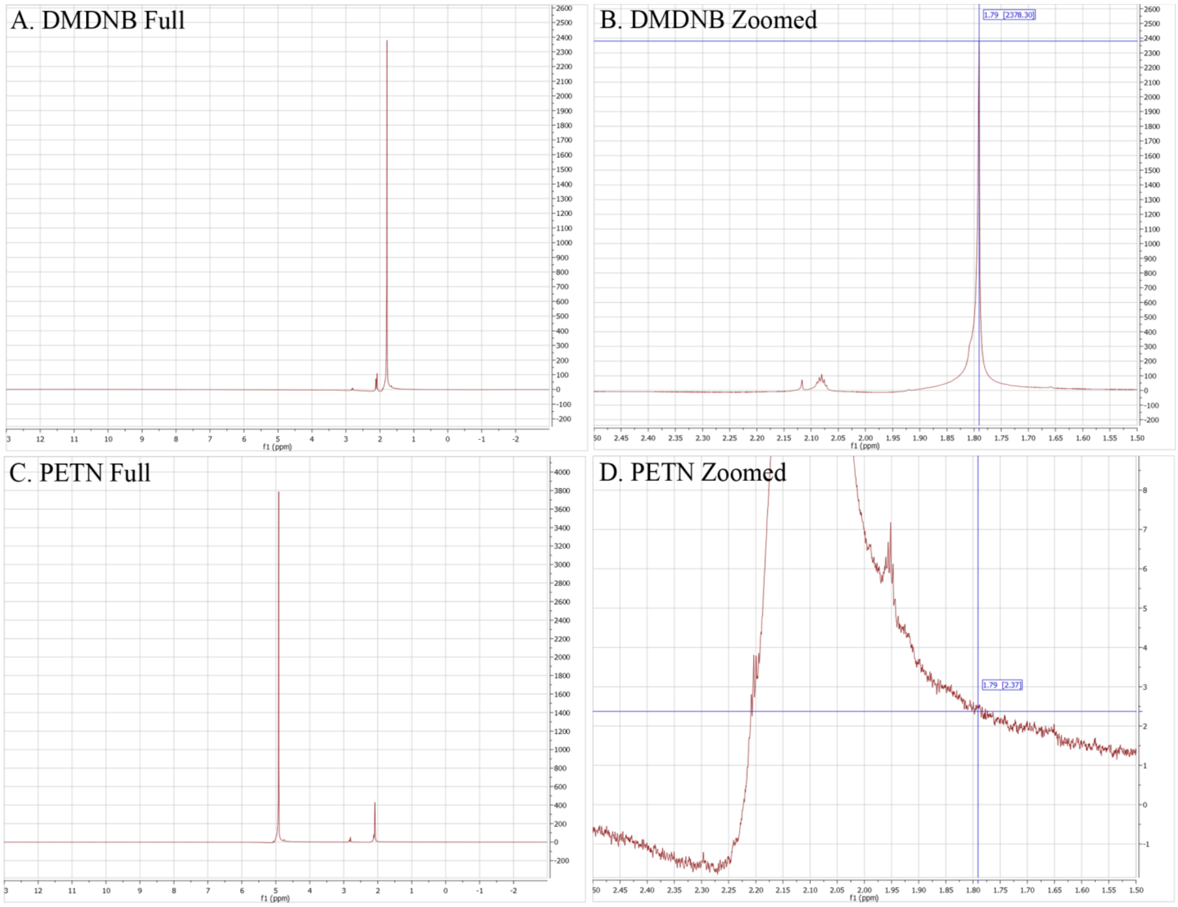

Figure S7. 1H-NMR spectra of DMDNB and PETN showing both a full range and zoomed in on the primary DMDNB peak at 1.79 ppm.

ESI References

1. *Convention of the Marking of Plastic Explosives for the Purpose of Detection*, International Civil Aviation Organization, Montreal, Canada, 1991.
2. *Public Law 104−132: Antiterrorism and Effective Death Penalty Act of 1996*, US Government, Washington, DC, 1996.
3. T. Nakamoto and H. Ishida, *Chem. Rev.*, 2008, **108**, 680-704.
